# Syndromic MEN1 parathyroid adenomas consist of both subclonal nodules and clonally independent tumors

**DOI:** 10.1007/s00428-023-03730-3

**Published:** 2024-01-20

**Authors:** Konstantin Bräutigam, Cédric Nesti, Philipp Riss, Christian Scheuba, Bruno Niederle, Tobias Grob, Annunziata Di Domenico, Maja Neuenschwander, Peter Mazal, Nastassja Köhn, Roman Trepp, Aurel Perren, Reto M. Kaderli

**Affiliations:** 1 Institute of Tissue Medicine and Pathology, University of Bern, Murtenstr. 31, 3008 Bern, Switzerland; 2 Department of Visceral Surgery and Medicine, Inselspital, Bern University Hospital, University of Bern, Bern, Switzerland; 3 Division of Visceral Surgery, Department of General Surgery, Medical University of Vienna, Vienna, Austria; 4 Department of Pathology, Medical University of Vienna, Vienna, Austria; 5 Department of General Surgery, Cantonal Hospital of Aarau, Aarau, Switzerland; 6 Department of Diabetes, Endocrinology, Nutritional Medicine and Metabolism, Inselspital, Bern University Hospital, University of Bern, Bern, Switzerland

**Keywords:** MEN1, Parathyroid, p27, Menin, Adenoma, NGS

## Abstract

Primary hyperparathyroidism with parathyroid tumors is a typical manifestation of Multiple Endocrine Neoplasia Type 1 (MEN1) and is historically termed “primary hyperplasia”. Whether these tumors represent a multi-glandular clonal disease or hyperplasia has not been robustly proven so far. Loss of Menin protein expression is associated with inactivation of both alleles and a good surrogate for a *MEN1* gene mutation. The *cyclin-dependent kinase inhibitor 1B* (*CDKN1B*) gene is mutated in MEN4 and encodes for protein p27 whose expression is poorly studied in the syndromic MEN1 setting.

Here, we analyzed histomorphology and protein expression of Menin and p27 in parathyroid adenomas of 25 patients of two independent, well-characterized MEN1 cohorts. The pattern of loss of heterozygosity (LOH) was assessed by fluorescence in situ hybridization (FISH) in one MEN1-associated parathyroid adenoma. Further, next-generation sequencing (NGS) was performed on eleven nodules of four MEN1 patients.

Morphologically, the majority of MEN1 adenomas consisted of multiple distinct nodules, in which Menin expression was mostly lost and p27 protein expression reduced. FISH analysis revealed that most nodules exhibited *MEN1* loss, with or without the loss of centromere 11. NGS demonstrated both subclonal evolution and the existence of clonally unrelated tumors.

Syndromic MEN1 parathyroid adenomas therefore consist of multiple clones with subclones, which supports the current concept of the novel WHO classification of parathyroid tumors (2022). p27 expression was lost in a large fraction of MEN1 parathyroids and must therefore be used with caution in suggesting MEN4.

**Supplementary Information:**

The online version contains supplementary material available at 10.1007/s00428-023-03730-3.

## Introduction

Multiple Endocrine Neoplasia Type 1 (MEN1) is a rare disorder with autosomal dominant inheritance, characterized by multiple tumors in endocrine organs [1]. Tumors of the parathyroid, the anterior pituitary gland, and the entero-pancreatic system are most frequent in MEN1 [1, 2]. MEN1 is clinically defined as the occurrence of tumors in two of the aforementioned organs or the occurrence of one tumor in combination with a first-degree relative having a known MEN1 disorder.

The first manifestation of the disease is most often primary hyperparathyroidism (PHPT) [3], i.e., hypercalcemia resulting from parathyroid tumors (single- versus multi-glandular disease), both of which are described in this context. As MEN1 patients usually have multi-glandular disease [4], the subtotal or total parathyroidectomy with autotransplantation is associated with the lowest risk of persistence and recurrence [5–7]. There appears to be a spectrum of disease aggression in MEN1. Histologically, neither clear-cut morphological nor recurrence-prompting features are reliably reproducible in MEN1 parathyroid tumors [8].

Multiple mutations have been described in the *MEN1* gene [9, 10] without a clear genotype–phenotype correlation [11]. MEN type 4 (MEN4), a more recently described MEN syndrome [12, 13], is caused by germline mutations in *cyclin-dependent kinase inhibitor 1B* (*CDKN1B*) [14] which encodes protein p27. MEN4 can mimic MEN1 [15], and *CDKN1B* mutations are implicated in the development of parathyroid adenomas as well [16–18].

The *MEN1* gene is a tumor suppressor gene, located on the long arm of chromosome 11 (11q13). It encodes the nuclear protein Menin [19], an epigenetic modifier, whose expression in MEN1 parathyroid adenomas is not extensively studied [20, 21]. Further, its role in predisposing to tumor formation is not yet fully understood [22]. The genotype does not always correspond to the same phenotype; the occurrence of tumors, the course of the disease, and the expression of symptoms vary even with the same mutation in the same family [23]. The genotype/phenotype mismatch is still under investigation, but there is some evidence that a variable degree of CpG island methylation of the *MEN1* gene, or its gene promoters could have an impact on the phenotype [24]. In duodenal and pancreatic MEN1-associated tumors and microtumors, it has been shown that the wild-type allele is inactivated most frequently by large deletions [25–27] and less frequently by point-mutations or promoter hypermethylation.

While MEN1-associated hyperparathyroidism traditionally was described as “primary hyperplasia”, increasing evidence points toward a multi-glandular neoplastic disease rather than a simple hyperplasia of the gland [28]. Some authors found a biological rationale for monoclonality [29], while others postulated independent genetic events within one enlarged parathyroid [30]. Finally, previous evidence by genetic probing suggested a coexistence of different clones in independent evolutionary stages [31].

The aim of this study was to further investigate the molecular background of syndromic MEN1 parathyroid adenomas using fluorescence in situ hybridization, next-generation sequencing with copy number analysis based on the relative number of reads and allelic imbalances, and immunohistochemistry to determine the potential presence of hyperplasia or of multiple independent clones and the amount of Menin loss. As MEN4 may be a clinical differential diagnosis of MEN1, p27 expression was also investigated to assess its value in suggesting a respective germline mutation.

## Material and methods

### Patient samples and clinical data

This retrospective study includes 25 MEN1 patients from the Medical University of Vienna and Bern University Hospital with surgical removal of parathyroid tumors between 1992 and 2022 (Table 1). *MEN1* germline mutations of 23 patients (92.0%) have been confirmed during the diagnostic genetic workup via sequencing in the respective institutions The spectrum of mutations is heterogeneous with only three recurrent mutations in two patients each (mutations summarized in Table S1).

**Table 1:** Cohort specification and histomorphology

	Vienna (*n* = 21)	Bern (*n* = 4)
Sex		
Male	12 (57.1%)	1 (25.0%)
Female	9 (42.9%)	3 (75.0%)
Age at surgery (years)	*x̄* = 41.3 (SD 9.1)	*x̄* = 33.0 (SD 7.5)
Nephro-/urolithiasis		
Yes	3 (14.3%)	1 (25.0%)
No	16 (76.2%)	2 (50.0%)
Not available	2 (9.5%)	1 (25.0%)
Kindred		
Positive	8 of 11 examined (72.2%)	1 of 1 examined (100%)
Negative	3 of 11 examined (27.8%)	0 of 1 examined (0%)
Preoperative osteodensitometry		
Normal	0	1 (25.0%)
Osteopenia	6 (28.6%)	2 (50.0%)
Osteoporosis	2 (9.5%)	0
Not available	13 (38.1%)	1 (25.0%)
Preoperative PTH (pg/ml)	*x̄* = 154.3 (SD 153.3)	*x̄* = 227.4 (SD 162.9)
Preoperative Ca (total, mmol/l)	*x̄* = 2.8 (SD 0.4)	*x̄* = 3.0 (SD 0.3)
Preoperative Ca (albumin-adjusted, nmol/l)	*x̄* = 2.7 (SD 0.4)	*x̄* = 2.9 (SD 0.1)
Preoperative albumin (g/l)	*x̄* = 43.1 (SD 1.5)	*x̄* = 37.5 (SD 4.9)
Preoperative creatinine (μmol/l)	*x̄* = 76.5 (SD 20.5)	50.0 (only one patient with data)
Preoperative 25-hydroxyvitamin D (nmol/l)	*x̄* = 48.6 (SD 23.1)	*x̄* = 30.8 (SD 1.7)
Surgical exploration		
OMIP	2 (9.5%)	0
UNE	1 (4.8%)	0
BNE	18 (85.7%)	4 (100%)
Parathyroid glands identified		
Four glands	15 (71.4%)	2 (50.0%)
Three glands	2 (9.5%)	2 (50.0%)
≤ 2 glands	2 (9.5%)	0
Not available	2 (9.5%)	0
Parathyroidectomy		
Total (min. four glands with autotransplantation)	12 (57.1%)	2 (50.0%)
Subtotal (three glands)	3 (25.0%)	2 (50.0%)
Less than subtotal (≤ 2 glands)	6 (28.6%)	0
Thymectomy^a^		
Yes	13 (61.9%)	0
No	8 (38.1%)	4 (100%)
Follow-up (months)	*x̄* = 85.6 (SD 76.4)	*x̄* = 101.5 (SD 162.9)
Persistence^b^	0	0
Recurrence^c^	3 (14.3%)	2 (50.0%)
Time to recurrence (months)	*x̄* = 170.3 (SD 101)	*x̄* = 83 (SD 106.1)
Histomorphology		
(Micro-)nodules per adenoma	x̄=2.6 (maximum 6)	x̄=8.3 (maximum 12)
Presence of septa	13 (61.9%)	4 (100%)
Rim of atrophic parathyroid tissue		
Yes	4 (19.0%)	1 (25.0%)
No	17 (81.0%)	3 (75.0%)
Cystic configuration	16 (76.2%)	4 (100%)
Main cell type		
Chief cell	16 (76.2%)	2 (50.0%)
Oxyphilic	4 (19.0%)	2 (50.0%)
Water clear	1 (4.8%)	0
Presence of a fibrous capsule	17 (81.0%)	2 (50.0%)

*BNE* bilateral neck exploration, *Ca* calcium, *OMIP* open minimally invasive parathyroidectomy, *PTH* parathyroid hormone, *SD* standard deviation, *UNE* unilateral neck exploration, *x̄* mean

^a^Thymectomy is defined as a complete resection of the (remaining) thymus

^b^Persistence designates biochemical hyperparathyroidism within the first 6 months postoperatively

^c^Recurrence designates biochemical hyperparathyroidism after six months of normal parathyroid function

Sex, age at surgery, presence of nephro-/urolithiasis, family history, preoperative osteodensitometry, parathyroid hormone (PTH), calcium, albumin, creatinine, and 25-hydroxyvitamin D levels; the type of surgical exploration, autotransplantation, number of parathyroid glands identified and removed, and synchronous thymectomy were extracted from electronic medical records at both participating institutions. In addition, the follow-up interval, persistence (biochemical hyperparathyroidism within the first 6 months postoperatively), and recurrence (biochemical hyperparathyroidism after 6 months of normal parathyroid function) were documented postoperatively.

**Fig. 1:**
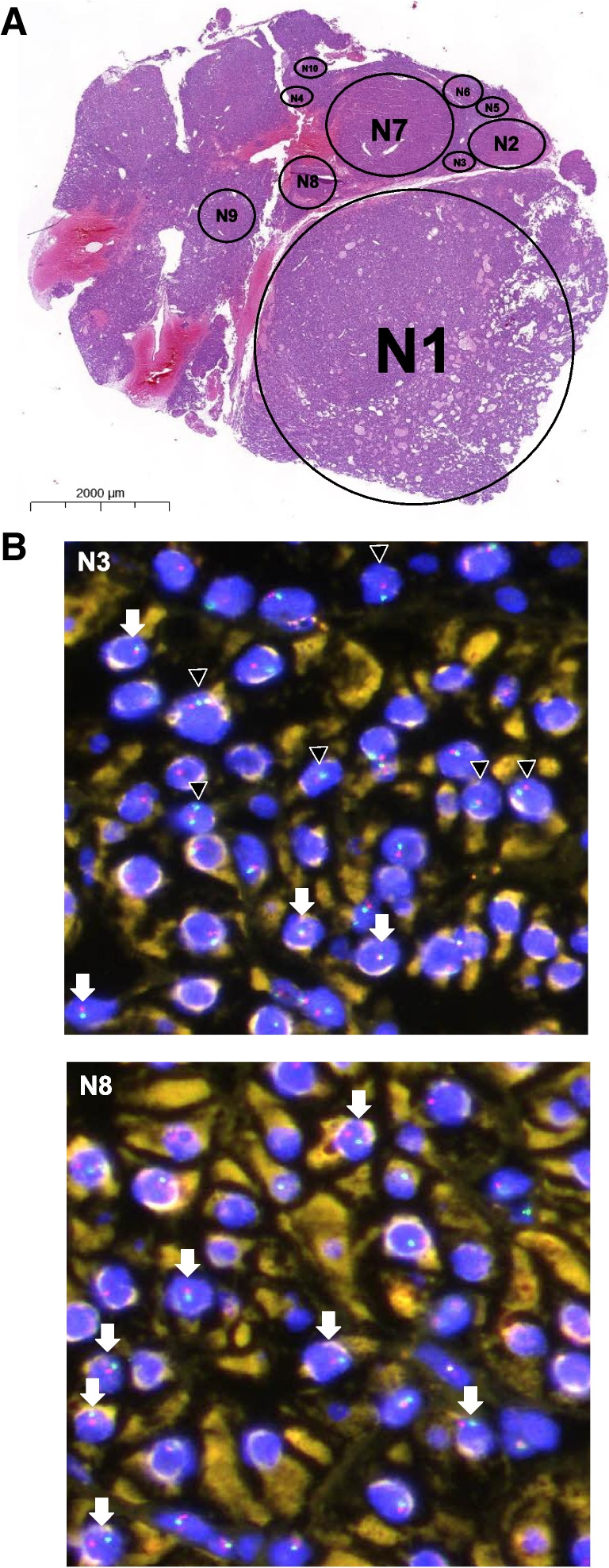
FISH analysis. **A** MEN1 parathyroid adenoma (H&E) consisting of ten distinct (micro)nodules (N) (extended version in Fig. S1). **B** FISH analysis of micronodules N3 and N8 reveals a heterogeneity of patterns; while N3 shows mostly *MEN1* loss of heterozygosity (LOH), N8 shows centromere 11 loss in most cells. Arrowheads indicate *MEN1* LOH. Arrows indicate centromere 11 loss. Red fluorophore indicates *MEN1* 11q13 locus and green fluorophore indicates *CEN11p* 11p11.12 locus

Morphologically distinct intra-glandular nodules and micronodules (< 5 mm, arbitrarily defined in analogy to microtumors of the pancreas [32]) were counted based on one hematoxylin–eosin (H&E) section per patient (compare Fig. 1).

The study was conducted in accordance with the Declaration of Helsinki (1964) and ethical approval was obtained from the local ethics committees in Vienna, Austria (reference number “2239/2019”) and Bern, Switzerland (“KEK-BE 105–2015”).

### Fluorescence in situ hybridization (FISH)

Sections of two parathyroid adenomas (MEN1 and non-MEN1) were hybridized using a commercial *MEN1* FISH probe (Abnova, Taipei, Taiwan; FG0040; *MEN1*/CEN11p FISH probe, genomic DNA, human origin) according to the manufacturer’s instructions (http://www.abnova.com/support/protocols.asp) and local standard operating procedures (e.g. in [33]). The formalin-fixed, paraffin-embedded (FFPE) FISH “PreTreatment Kit 1” (Abnova) was used for pretreatment. Using 4′,6-diamidino-2-phenylindole (DAPI) as a counterstain, the Texas red fluorophore was used to label the *MEN1* 11q13 locus (approximately 500 kb), and the green (FITC) fluorophore to label the *CEN11p* 11p11.12 locus (approximately 630 kb). Tissue regions were photographed with an Olympus XM10 monochrome digital microscope camera using an Olympus BX61VS microscope system (Olympus, Tokyo, Japan). Up to seven regions were examined in each morphologically distinct intra-glandular nodule (“N”) (Fig. 1A, Fig. S1). Per region, all signals per cell were visually counted by one pathologist (KB).

### Next-generation sequencing (NGS)

NGS has been performed in parathyroid adenomas of four MEN1 patients. Genomic DNA was extracted from 1-mm tissue punches of two to three nodules per patient (Fig. S2) and sequenced using the “True Sight Oncology (TSO) 500 DNA” panel (Illumina, San Diego, CA) [34] according to manufacturer instructions with adequate coverage depth. The allelic imbalance ratio was calculated for each single nucleotide polymorphism (SNP) from 0 to 1. Tumor cell content per region has been at least 70%. Significant copy number alterations, i.e., large chromosomal aberrations, were identified based on the combination of the number of reads and allelic frequency using in-house standards. Areas of (morphologically) diffuse hyperplasia (with the inclusion of fat cells) were too small to be microdissected and could not be sequenced.

### Immunohistochemistry (IHC)

**Fig. 2:**
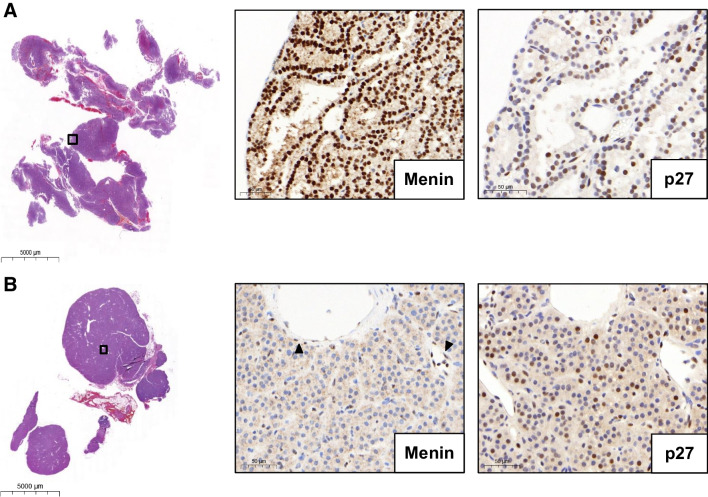
Menin and p27 immunohistochemistry. **A** A non-MEN1 parathyroid adenoma control shows retained and strong nuclear MEN1 expression. Nuclear p27 expression is retained in most cells (> 60%). **B** An exemplary MEN1 parathyroid adenoma with loss of nuclear menin expression (preserved expression in the endothelium; arrowheads). Focal absence of nuclear p27 expression

Sections (2.5 µm) were deparaffinized in dewax solution (Leica Biosystems, Muttenz, Switzerland) and rehydrated. p27 IHC was performed on a BOND RX (Leica), and Menin IHC on a benchmark automated immunostainer (Ventana, Roche, Arizona, USA). Slides were scanned on a Panoramic 250 Flash scanner (3DHistech, Budapest, Hungary). Staining conditions were as follows: Menin (A300–105A, Bethyl Lab, Fortis Life Sciences, Massachusetts, USA), dilution 1:800, retrieval—citrate buffer, 32 min, 100 °C; antibody incubation—60 min at 37 °C. p27 (SX53G8; #427 M, Cell Marque, Sigma-Aldrich, California, USA), dilution 1:500, retrieval—citrate buffer, 30 min, 100 °C; antibody incubation—15 min at room temperature. Immunohistochemistry was evaluated by two pathologists (KB, AP) on whole slides. For both markers, only nuclear staining was considered positive. According to previous literature [35], p27 is expressed on an average of approximately 60% of neoplastic cells in parathyroid adenomas. Therefore, we defined reduced nuclear expression as expression in less than 60% but more than 20% of neoplastic cells. Menin (p27) expression was considered as lost (absent) only in the presence of an internal positive control. All non-neoplastic cells, i.e., mainly endothelium and stroma (on slide), and a non-MEN1 adenoma were used as positive controls (Fig. 2A). Negative on-slide controls were not available.

### Statistics

Statistical analyses were performed using SPSS version 28.0 (SPSS Inc., Chicago, IL, USA). Two-sided Pearson’s *χ*^2^ tests were used to calculate contingency tables; *p* < 0.05 was considered statistically significant. Correlation analyses were conducted using Spearman’s rho coefficients (*ρ*).

## Results

The 25 MEN1 patients included had a mean age at surgery of 40.0 years (SD 9.3) (Table 1). Surgical procedures comprised 14 (56.0%) total, five subtotal (20.0%), and six (24.0%) less than subtotal parathyroidectomies with 13 (52.0%) synchronous thymectomies. The mean follow-up was 88.2 months (SD 76.6).

### Syndromic MEN1 adenomas consist of multiple morphologically distinct nodules

While parathyroid hyperplasia shows diffuse, nodular, or mixed growth patterns [36], typical morphological stigmata for MEN1 parathyroid adenomas have not been reported. Reliable differentiation from secondary hyperplasia is difficult, if not impossible, based on histomorphology [37]. Importantly, the majority of MEN1 adenomas (16/25 [64.0%]) in our cohorts revealed at least one distinct large nodule with up to 11 additional smaller (micro)nodules (Table 1). Only four patients (16.0%) did not show any evidence of intra-glandular (micro)nodules.

In total, 17/25 (68.0%) MEN1 adenomas showed at least one fibrous septum, and 19/25 (76.0%) adenomas were at least partially encapsulated (Fig. 1A). The majority were composed mainly of chief cells (18/25 [72.0%]). 5/25 (20.0%) MEN1 adenomas showed at least a focal rim of adjacent non-neoplastic parathyroid tissue. Focal endocrine atypia in terms of larger irregular hyperchromatic nuclei was found in 3/25 (12.0%) MEN1 adenomas. In 15/25 (60.0%) samples focal remaining fat was observed. All 25/25 (100%) MEN1 adenomas showed sparse stromal fat, at least focal cystic configurations, and an absence of calcifications, necrosis, or invasion of adjacent skeletal muscles or vessel invasion.

### FISH suggests individual molecular states in syndromic MEN1 adenomas

To further investigate *MEN1* loss of heterozygosity (LOH), we compared a MEN1 parathyroid adenoma (“Bern 3”, c.563G > C/p.W188S mutation) with a non-MEN1 parathyroid adenoma using a *MEN1* specific and centromere 11 (C11) fluorescence in situ hybridization (FISH) probe.

In the examined non-MEN1 adenoma, three intra-glandular micronodules could be distinguished. The MEN1 adenoma showed ten nodules (Fig. 1A, Fig. S1). Even though there were many non-interpretable signals, we consistently noticed *MEN1 *loss of heterozygosity (LOH) and/or centromere 11 in the micronodules of the MEN1 adenoma (Fig. 1B). Quantitatively, centromere 11 loss and *MEN1 *LOH were much more frequent in the MEN1 adenoma compared to the non-MEN1 adenoma.

In immunohistochemistry, all evaluable (micro)nodules showed complete loss of Menin and reduced p27 protein expression.

### Next-generation sequencing (NGS) reveals both clonally distinct tumors and subclonal evolution

NGS confirmed the known *MEN1* germline mutations in all investigated (*n* = 4) patients and nodules (*n* = 11). No additional driver mutations could be detected in any of the sequenced regions. All the investigated regions showed evidence of clonality and not of hyperplasia, by evidence of LOH of the *MEN1* wild-type allele (Fig. S2, Table 2). In two parathyroids, we observed different second hits of the *MEN1* region, i.e., copy number–neutral (CNN) LOH and biallelic loss, highly suggestive of the coexistence of clonally independent tumors (Fig. S2A and S2D). We also found evidence of subclonal evolution, i.e., some intra-glandular nodules have acquired additional aberrations (Fig. S2C). One adenoma shows the same aberrations in both investigated nodules (Table 2).

**Table 2:**
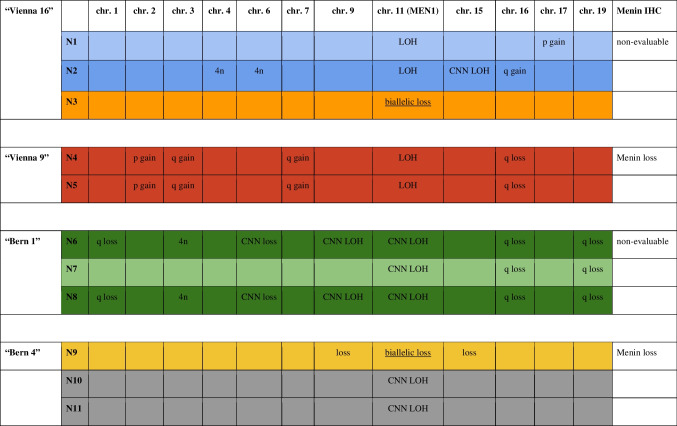
Specific and shared copy number alterations per patient and nodule (N) (plots in Fig. S2)

*4n* tetraploidy; *chr.* chromosome; *CNN* copy number neutral; *IHC* immunohistochemistry; *LOH* loss of heterozygosity

The morphology of subclonal or clonal nodules was not significantly different. Some nodules showed cystic transformation, and other regions were slightly more oncocytic (Fig. S2) without a clear correlation with the genotype.

Tumor mutational burden (TMB) was low in all eleven investigated nodules (each nodule < 4 somatic mutations per megabase), and all were microsatellite stable.

### Immunohistochemistry for Menin and p27

In this study, we observed a complete loss of Menin expression in MEN1 parathyroid adenomas (Fig. 2, Fig. S3, Table 3) in all regions evaluable. 9/25 (36.0%) patients showed positive internal controls. Nuclear Menin expression was lost regardless of the type of mutation present. Unfortunately, only 2/25 (8.0%) samples were evaluable in the entire tissue section. 7/25 (28.0%) samples were partially evaluable, mostly in the periphery of the tissue section. As the probes were collected over a time span of 20 years, 16/25 (64.0%) of the samples were not evaluable due to differing preanalytical conditions.

**Table 3:** Interpretation of Menin and p27 immunohistochemistry

	Menin		p27
Bern	1/4 (25.0%) evaluable	Loss	2/4 (50.0) reduced
	1/4 (25.0%) partially evaluable	Loss	2/4 (50.0) absence of expression
	2/4 (50.0%) non-evaluable		
Vienna	1/21 (4.8%) evaluable	Loss	8/21 retained (38.1%)
	6/21 (28.6%) partially evaluable	6/6 loss (100.0%)	9/21 reduced (42.9%)
	14/21 (66.7%) non-evaluable		4/21 absence of expression (19.0%)

p27 was reduced in 11/25 (44.0%) samples. Six of 25 (24.0%) samples showed a complete absence of expression, while expression was retained in 8/25 (32.0%) of the samples. Among the samples with at least partially evaluable Menin expression, p27 expression was reduced in four (44.4%) and retained in five (55.6%) samples, respectively.

### Recurrent PHPT correlates with younger patient age and preoperative PTH levels

Little is known about risk factors for the recurrence of PHPT in MEN1 patients. In all, 5/25 (20.0%) patients had recurrent PHPT, the earliest 8 months after surgery. Neither the number of identified glands (*ρ* = 0.092; *p* = 0.675) nor the number of removed glands (*ρ* = 0.12; *p* = 0.954) correlated with recurrent PHPT. However, we found a significant correlation between recurrent disease with younger patient age (*ρ* =  − 0.486; *p* = 0.014) and higher preoperative PTH levels (*ρ* = 0.571; *p* = 0.004). The number of intra-glandular (micro)nodules did not show a significant correlation with clinical features or outcomes, respectively.

## Discussion

MEN1 parathyroid adenomas consist of multiple morphologically distinct nodules and micronodules. We observed clonal molecular changes in all nodules, consequently we did not detect nodular hyperplastic areas. Therefore, we provide further evidence that parathyroid tumors in MEN1 are multiple monoclonal tumors and not hyperplastic changes, supporting the nomenclature of multi-glandular adenomas as suggested in the novel WHO classification of “*Endocrine and Neuroendocrine Tumours*” (5th edition, 2022) [38].

To the best of our knowledge, multiple individual clones in MEN1 parathyroid adenomas have not been robustly proven to date. Using restriction fragment length polymorphism of the x chromosome–linked PGK gene and differential methylation of cytosine residues in 14 non-MEN1 patients, nodular parathyroid hyperplasia was suggested to be monoclonal and diffuse parathyroid hyperplasia to be polyclonal [29]. Genetic analysis of 14 MEN1 parathyroid adenomas revealed evidence of monoclonality as evidenced by allelic loss [31]. It has been hypothesized that inactivation of the *MEN1* gene leads to tumor initiation and progression. FISH is a reliable method to detect LOH [39]. We show evidence of clonality in all examined (micro)nodules using FISH in one patient. The nodules showed *MEN1* LOH, and in all those nodules, we could prove *MEN1* inactivation by loss of immunohistochemical Menin expression with retained immunoreactivity in internal positive controls. Our observations are comparable to those in tumors of the endocrine pancreas where LOH was shown for tumors but not for islet hyperplasia in MEN1 [32]. NGS confirmed all known germline *MEN1* mutations, no additional oncogenic driver mutations were detected in > 500 investigated genes. We found evidence for both subclonal evolution as well as clearly clonally unrelated nodules in the same parathyroid indicated by different patterns of *MEN1* LOH and chromosomal imbalances. Morphologically, we could not distinguish these molecular patterns. The chromosomal imbalances observed in clones and subclones have been previously described in sporadic parathyroid adenomas, i.e., copy number neutral LOH of *MEN1* [10]; 1q and 15q loss [40]; partial loss of chromosome 9, 3q, 16q, and 17p gains [41]; as well as abnormal ploidy [42]. One of our patients demonstrated a reproducible 19q loss, while only 19q gains have been described in (“large”) parathyroid tumors [41]. Interestingly, there was little concordance regarding the observed aberrations (only 16q loss observed in two patients), underlining not only an intra-tumoral but also inter-patient genetic heterogeneity, indicating different clonal evolutions in individual patients.

mRNA analyses have demonstrated that Menin expression is downregulated in MEN1 parathyroid tumors [43]. This is consistent with immunohistochemistry, where Menin expression was lost in the nucleus of tumor cells. The reported sensitivity and specificity of Menin immunohistochemistry for MEN 1 are 86% and 87%, respectively [20]. Its interpretation is difficult because MEN1 does not necessarily correlate with a complete loss of protein expression [37, 44]. Here, Menin immunohistochemistry was only evaluable in the entire tissue of two and at the well-fixed periphery of seven samples. A recent study [21] suggests that Menin immunohistochemistry is a useful screening tool for MEN1 especially when genetic testing is inconclusive or inaccessible. The authors describe nuclear Menin loss in parathyroid tumors in 16/16 (100.0%) MEN1 patients of their single-center cohort, with MEN4 not taken into account. Our findings are in line with this, as we could confirm Menin loss in all, at least partially evaluable, samples. However, while MEN1 stainings work reproducibly with recently standardized pre-analytics, in this archival series over many years, reliable results could be detected only in 36.0% of cases.

The absence of p27 protein expression has been described in primary hyperparathyroidism and *MEN1* mutant parathyroids [45, 46]. Here, immunohistochemistry suggested that the p27 protein is mostly downregulated in MEN1 with a concomitant absence of expression in MEN1 patients. In general, parathyroid adenomas show reduced nuclear p27 positivity compared to hyperplasia or normal parathyroid glands [35]. As p27 can be (partially) lost in sporadic parathyroid adenomas [35, 37, 45], interpretation is difficult and does not prove a syndromic background. MEN4 is clinically a differential diagnosis of MEN1, our results show that loss of p27 expression cannot be used as potential evidence for this disease as p27 is frequently negative in MEN1 parathyroids as well.

Finally, the clinical management of MEN1 parathyroid adenomas is an ongoing debate [7]. In this study, we observed few significant clinicopathologic correlations. Histologically, a rim of adjacent atrophic parathyroid tissue is described to occur in the majority of (non-syndromic) parathyroid adenomas [47–49]. Here, only a fifth of our MEN1 samples showed this histomorphological feature.

Our study has several limitations. First, although we study a large cohort of MEN1 parathyroid adenomas from two academic centers, our study relies mainly on archived FFPE material. Although comparatively large, the number of patients included is still small for statistical analysis. Second, the Menin antibody used was only satisfactory on parts of the sections of in total of seven (28.0%) patients. Differences in fixation and processing procedures may explain the heterogeneity of protein expression, as our experience is much better in samples processed in recent years. Moreover, reproducible cutoffs for p27 immunohistochemistry in MEN1 have not been defined so far with conflicting observations. Third, some micronodules were lost in deeper sections, making direct comparison of staining difficult. Fourth, the FISH procedure led to relatively weak signals (frequently not interpretable) and could only be performed on a single MEN1 adenoma. Therefore, we interpret the results of our FISH analysis with caution. Fifth, *CDKN1B* germline mutation analysis to rule out MEN4 has not been performed, but respective mutations were excluded in the somatic NGS analysis. Copy-number calls derived from relative read numbers are semi-quantitative and insensitive for short genetic alterations.

In summary, we provide molecular evidence that enlarged MEN1 parathyroids consist of multiple subclones as well as clonally unrelated tumors in an autonomous evolutionary trajectory and that the term “primary hyperplasia” is therefore biologically incorrect. Complete nuclear Menin loss is frequent in MEN1 parathyroids, which may be of help in separation from secondary hyperparathyroidism. Ultimately, as p27 protein expression was at least partially absent in the majority of our MEN1 samples, the use of p27 immunohistochemistry cannot reliably suggest MEN4 in patients.

### Supplementary Information

Below is the link to the electronic supplementary material.

Supplementary Figure S1 (Micro-)Nodules in a MEN1 parathyroid adenoma. **A**: Ten morphologically identifiable nodules (N1) and micronodules (N2 to N10); overview. **B**: (Micro-)Nodules in high resolution. (PDF 4686 KB)

Supplementary Figure S2 Next-generation sequencing (NGS) of eleven nodules (“N”) in four MEN1 patients. **A** (patient “Vienna 16”, Table S1): N3 demonstrates biallelic MEN1 loss and is probably a different tumor, while N1 and N2 show conventional MEN1 LOH. N1 and N2 have different gains supporting subclonal evolution. **B** (patient “Vienna 9”): N4 and N5 demonstrate the same gains and losses, and represent most probably the same tumor. **C** (patient “Bern 1”): All three nodules share a CNN LOH of chromosome 11, as well as 16q and 19q losses. N6 and N8 have identical additional aberrations, suggesting N7 to be the primary clone with subclonal evolution in N6 and N8. Interestingly, N7 and N8 have cystic components. **D** (“Bern 4”): N9, N10 and N11 show different types of MEN1 inactivation supporting the concept of different tumors. In addition, N9 has further losses, N10 and N11 do not have.
*Bars (y-axis)*: number of reads; CNN: copy number neutral; LOH: Loss of heterozygosity; *scatter plot*: allelic frequency/single nucleotide polymorphisms (SNPs); *x-axis*: chromosomes (on the left q-arm, on the right p-arm). (PDF 7888 KB)

Supplementary Figure S3 Menin loss in a MEN1 parathyroid adenoma. **A**: MEN1 parathyroid adenoma (H&E), on the right Menin immunohistochemistry (overview). **B**: Loss of Menin expression in a larger clone, adjacent non-neoplastic parathyroid tissue (arrow) and intermingled non-neoplastic cells (arrowhead) with retained Menin expression. *Inset*: 40x magnification.(PDF 287 KB)

Supplementary Table S1 (DOCX 16.1 KB)

## Data Availability

The datasets used and/or analyzed during the current study are available from the corresponding author upon reasonable request.
