# Supplementary figures and images for: Syndromic MEN1 parathyroid adenomas consist of both subclonal nodules and clonally independent tumors
Source: Virchows Arch. 2024 Jan 20;484(5):789–98. doi: 10.1007/s00428-023-03730-3 (PMC11106174; doi:10.1007/s00428-023-03730-3)

A

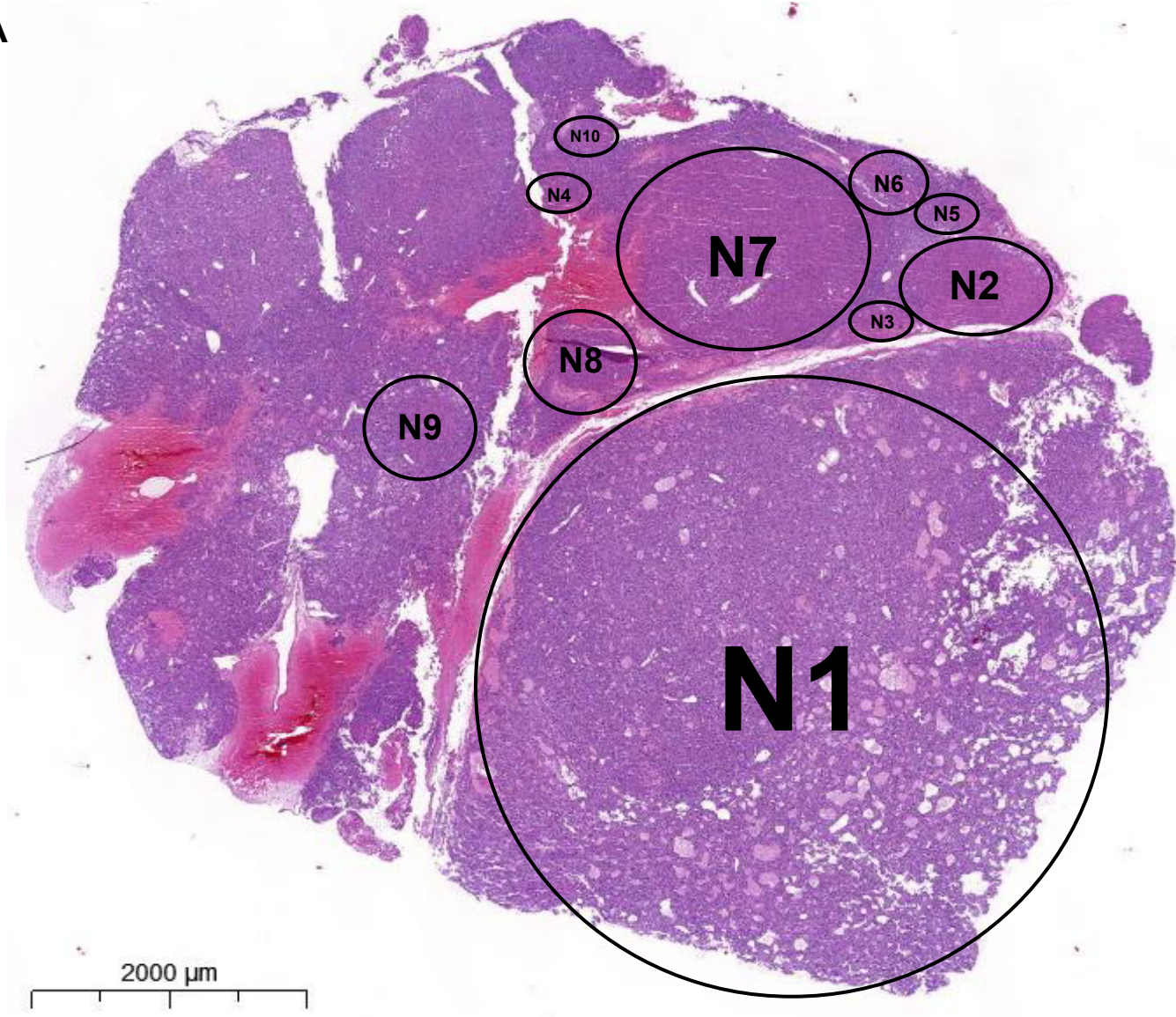

B

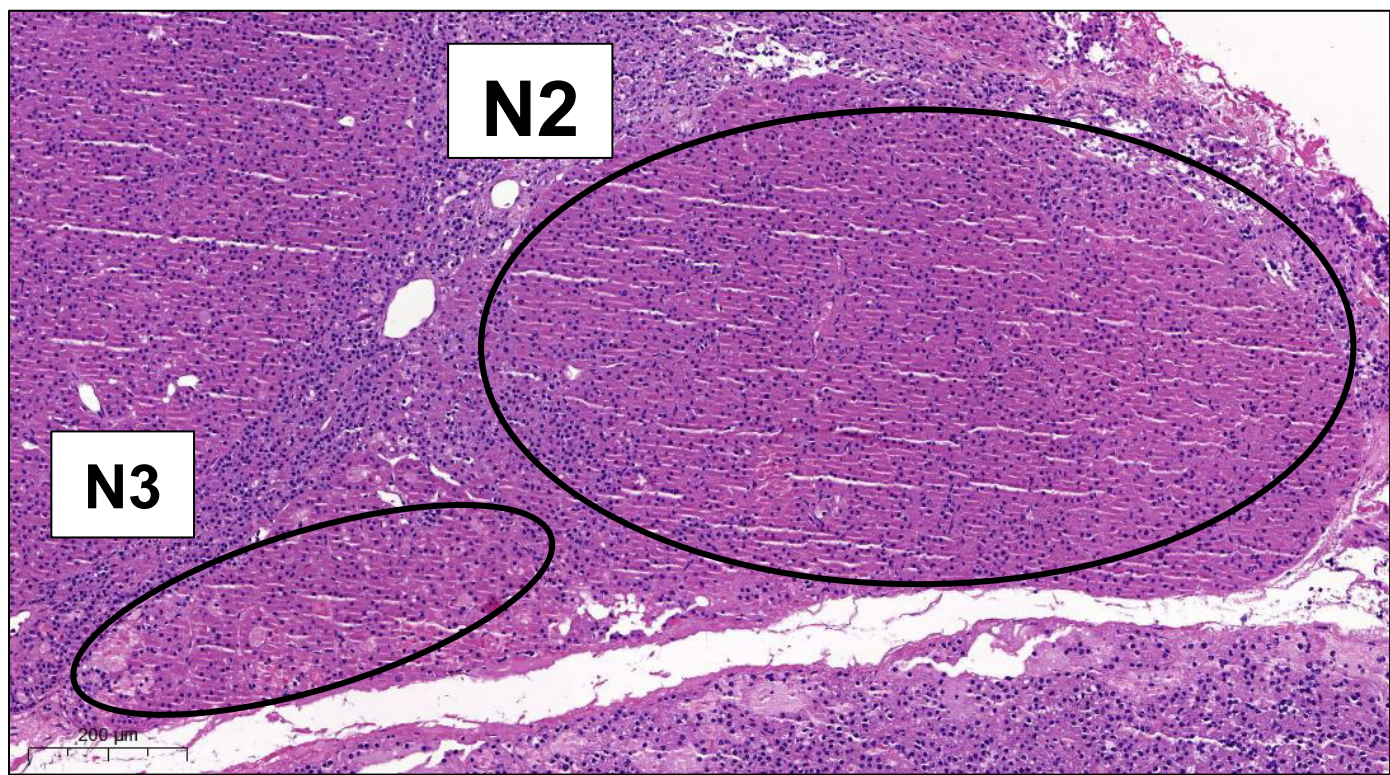

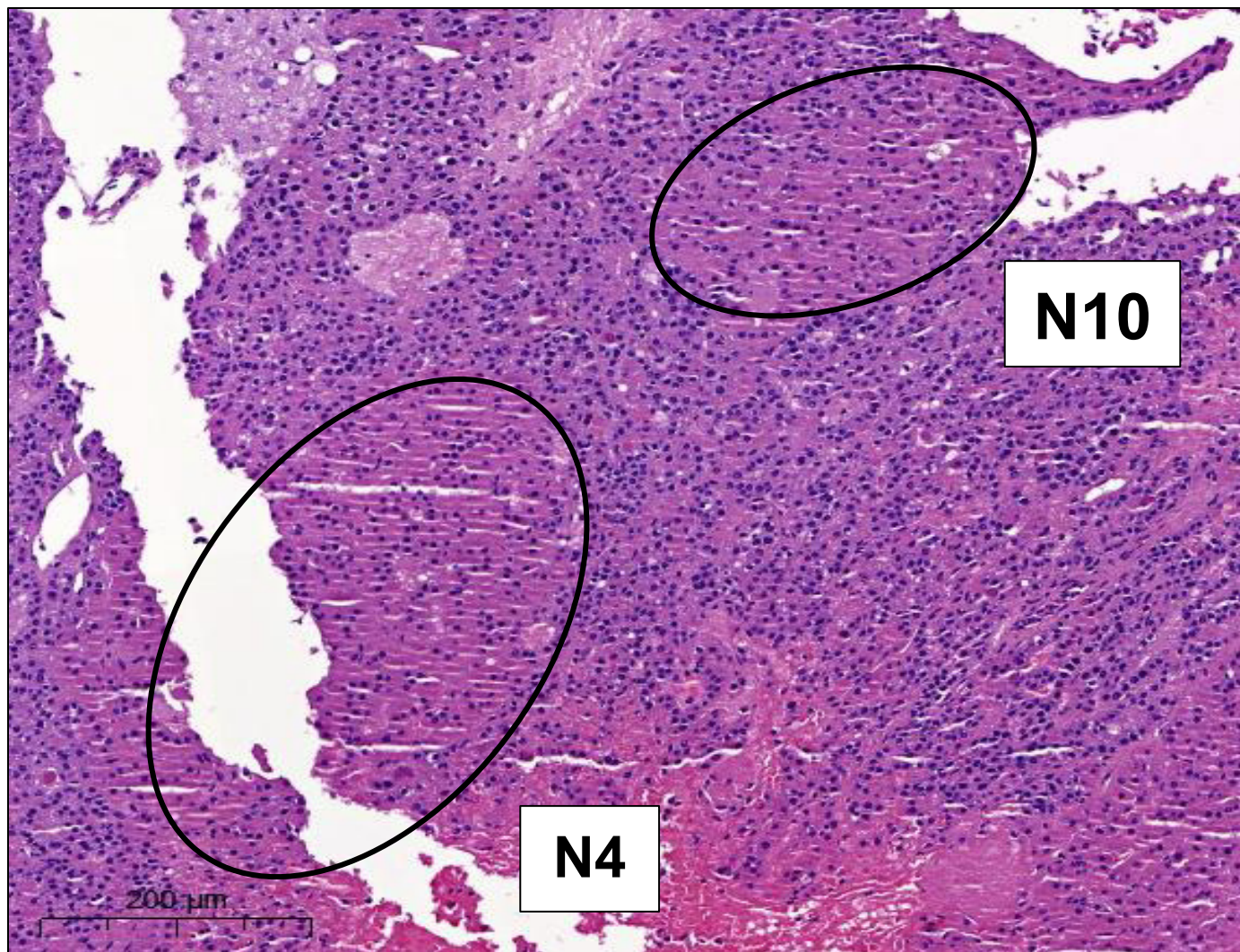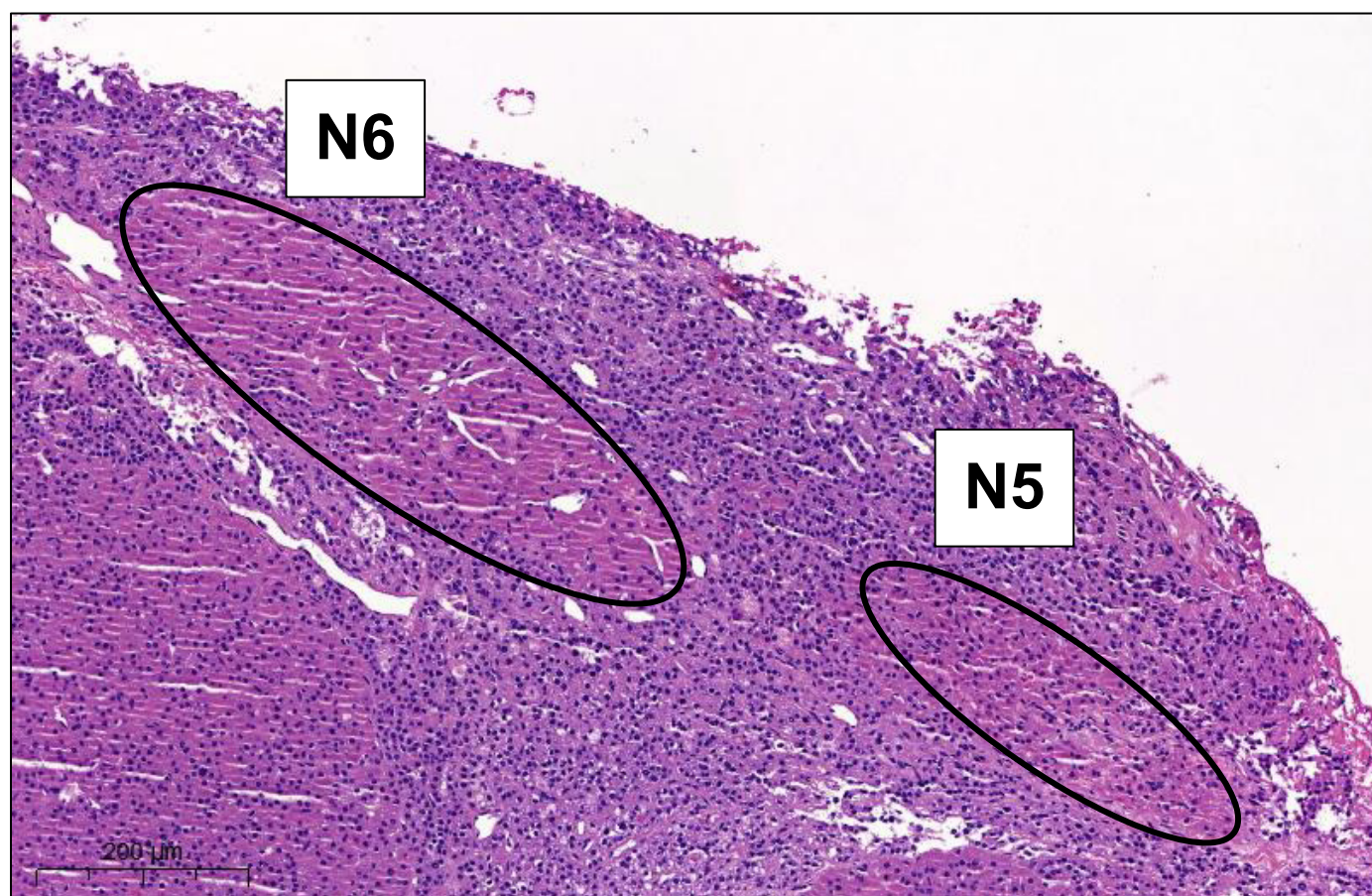

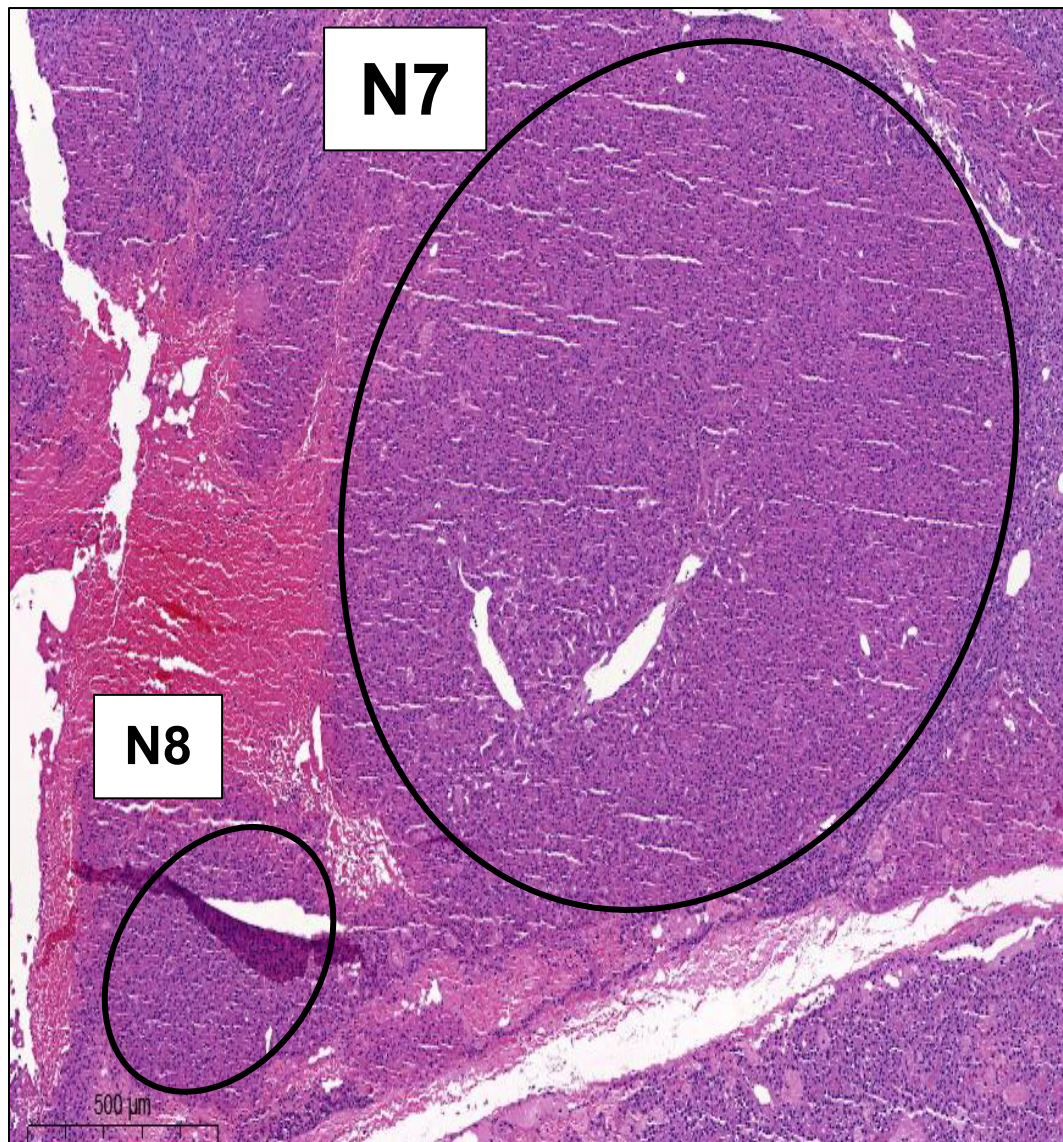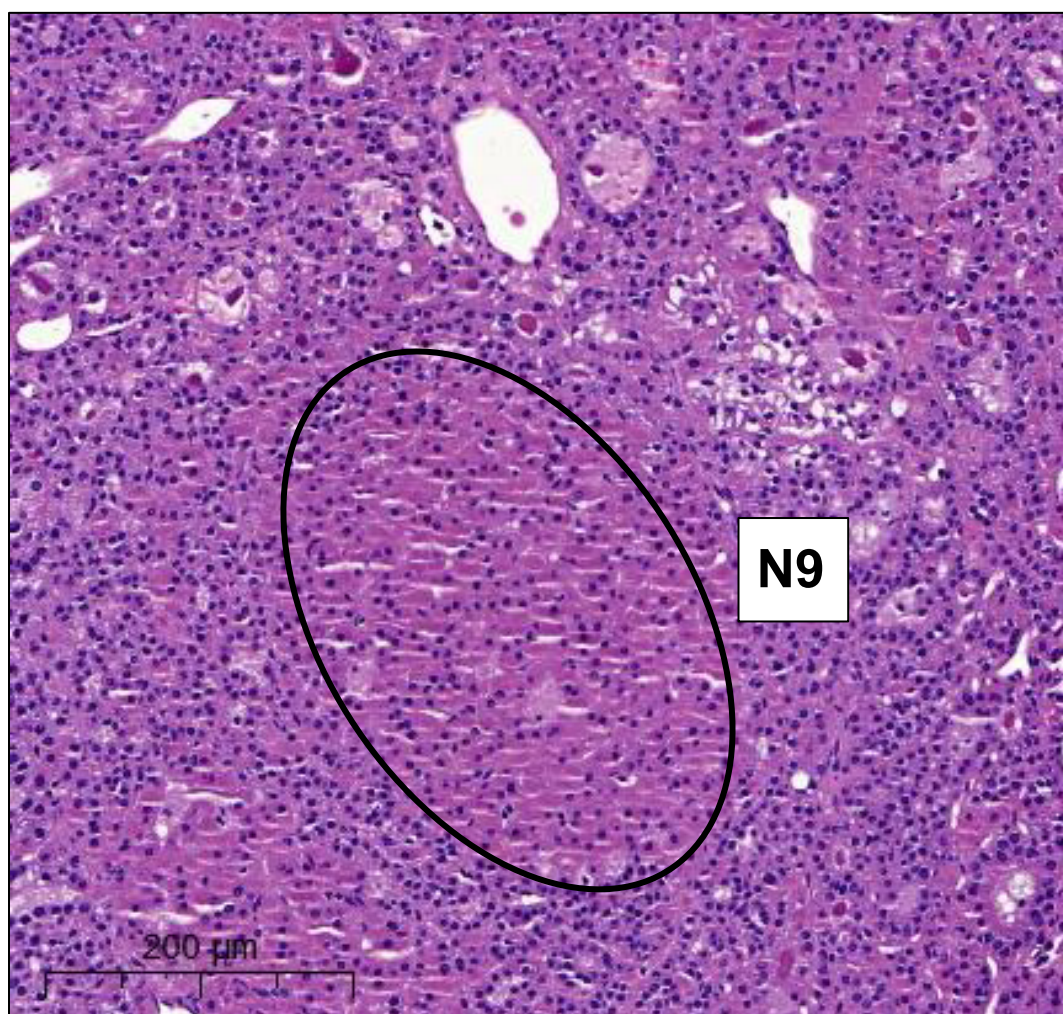

Supplement: Supplementary file 1 — (Micro-)Nodules in a MEN1 parathyroid adenoma. A: Ten morphologically identifiable nodules (N1) and micronodules (N2 to N10); overview. B: (Micro-)Nodules in high resolution. (PDF 4686 KB) [file 428_2023_3730_MOESM1_ESM.pdf]

A

N1

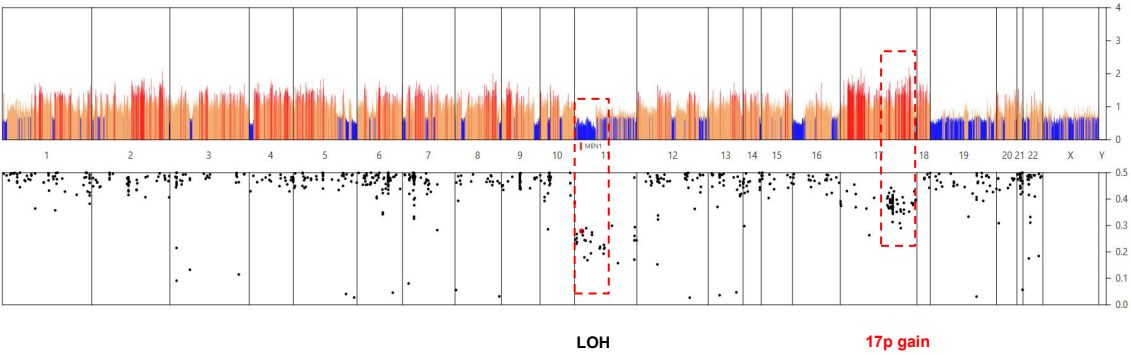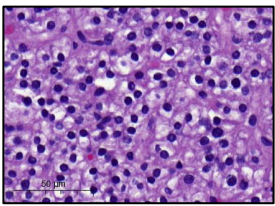

N2

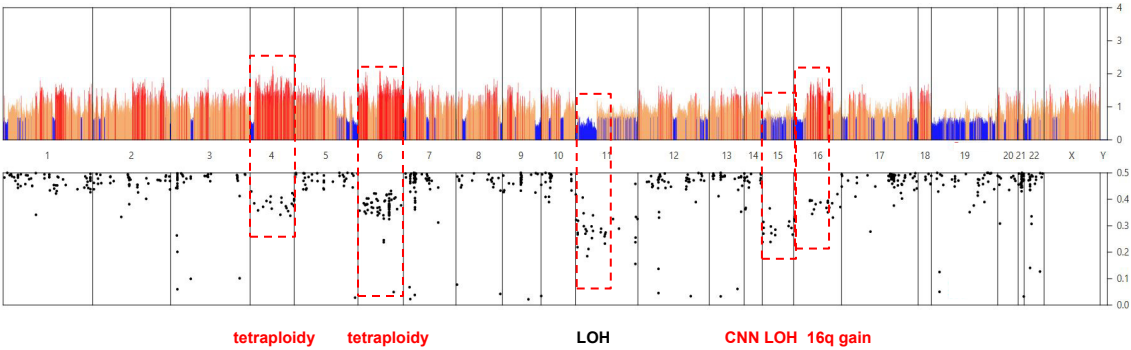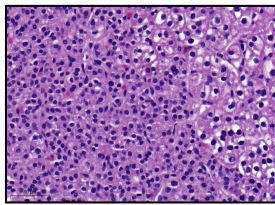

N3

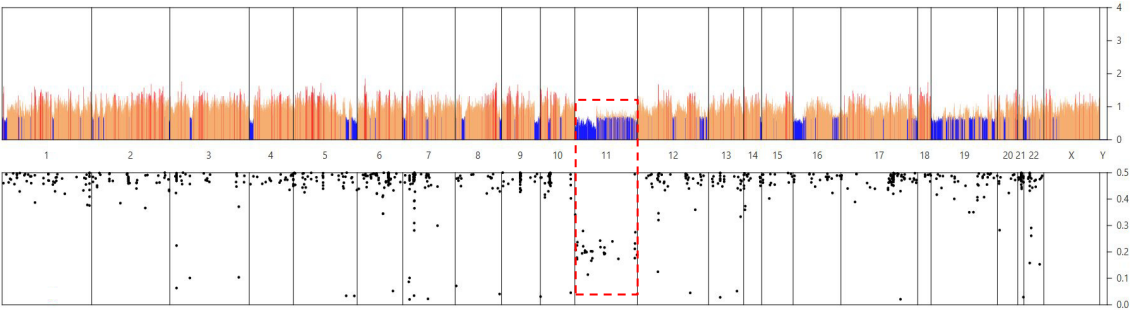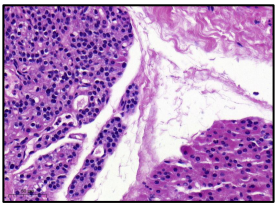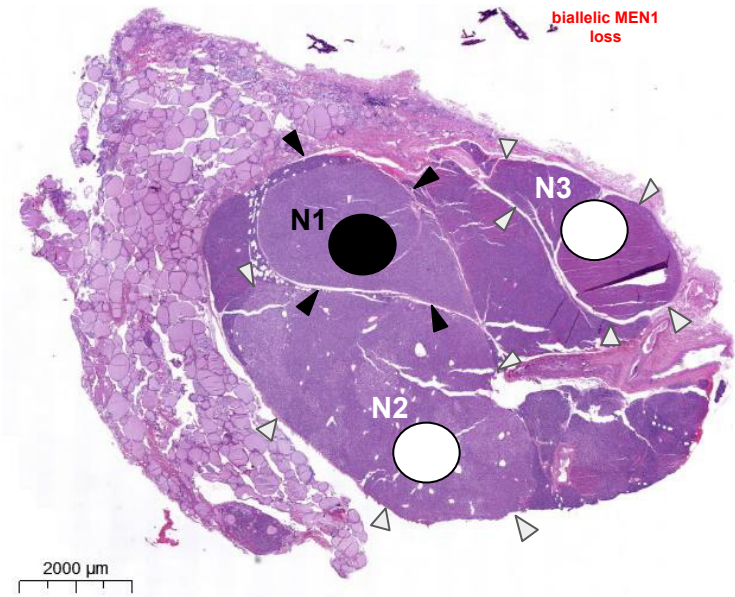

B

N4

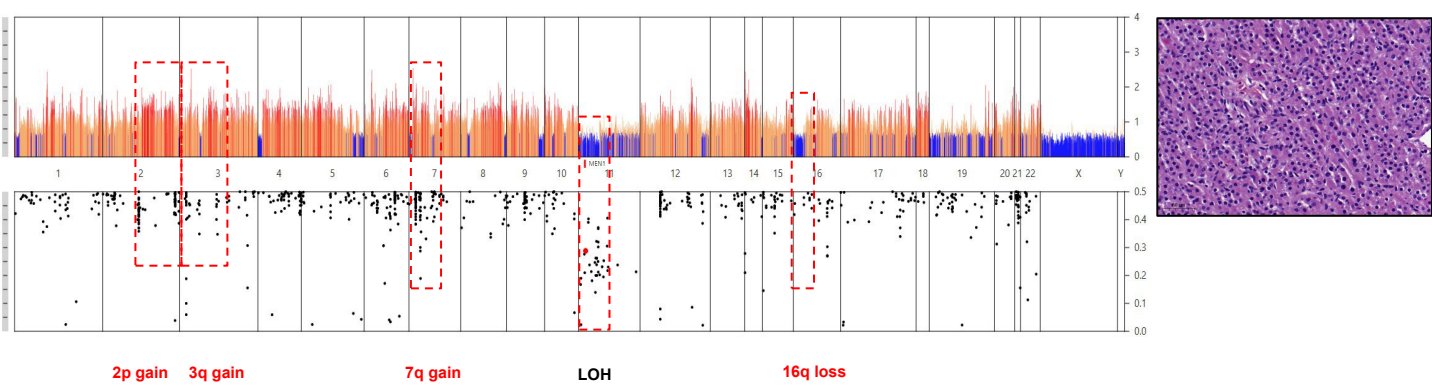

N5

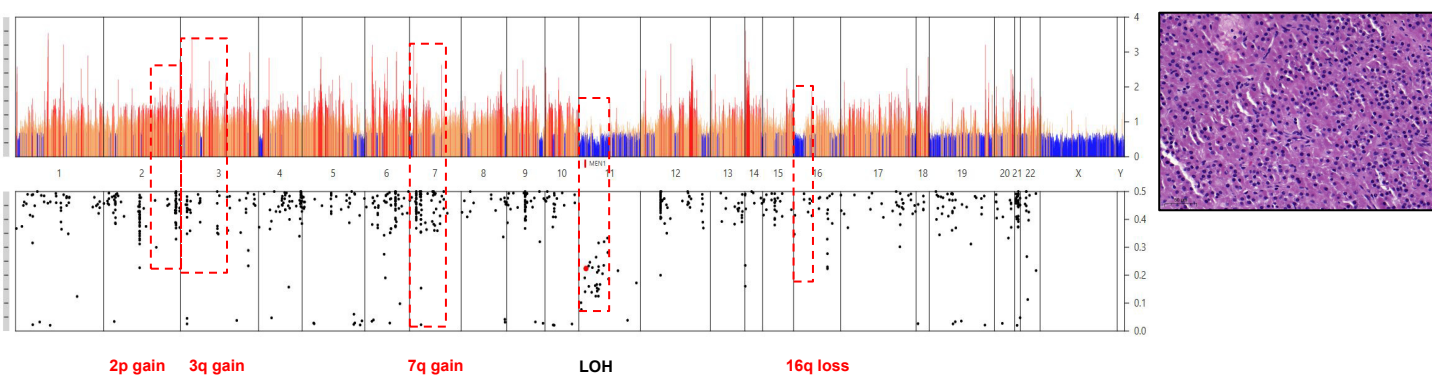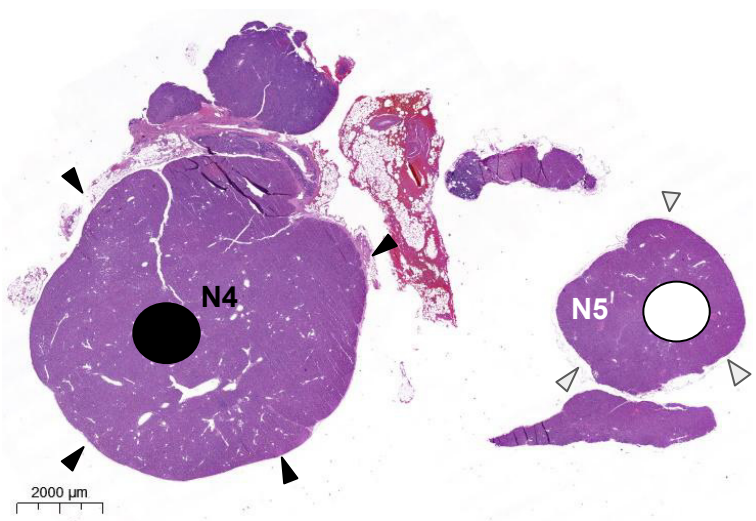

C

N6

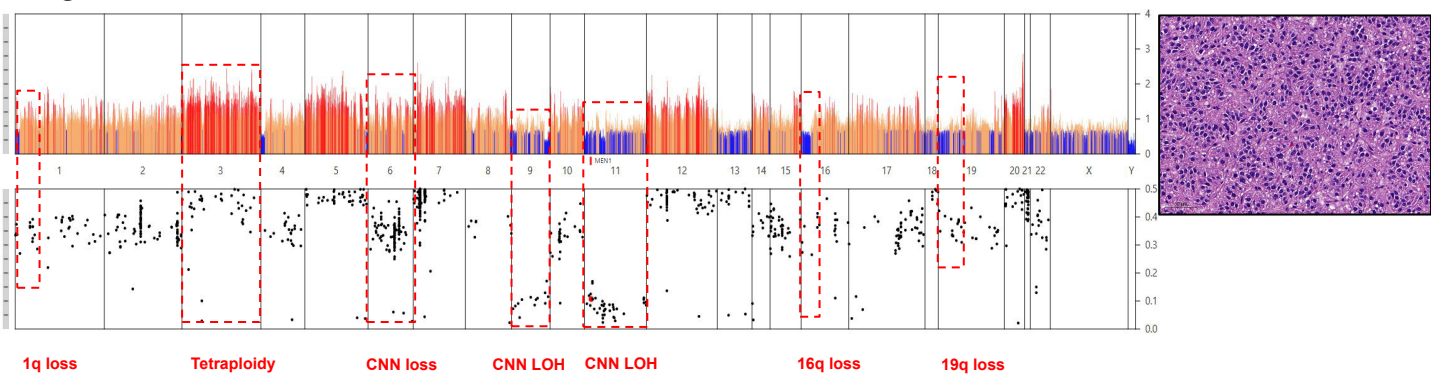

N7

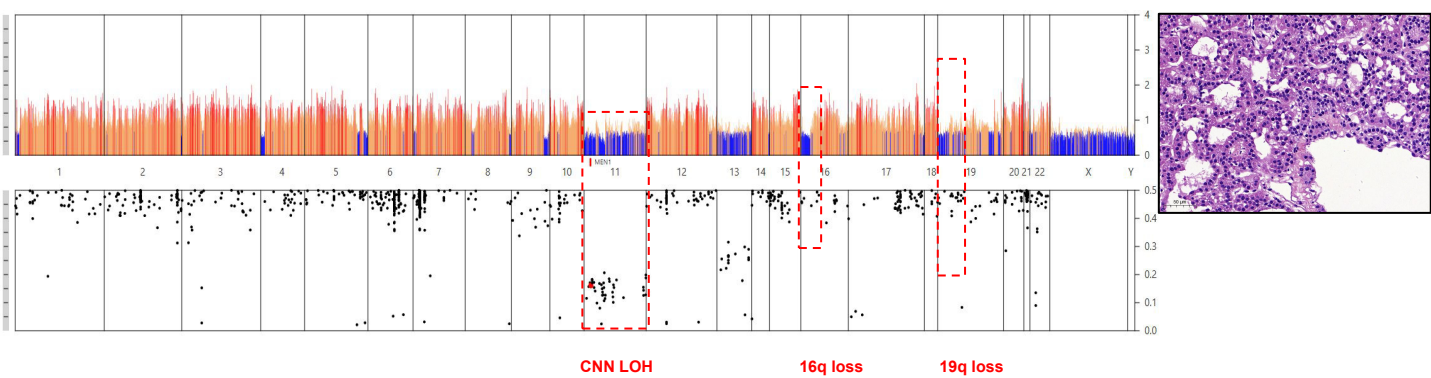

N8

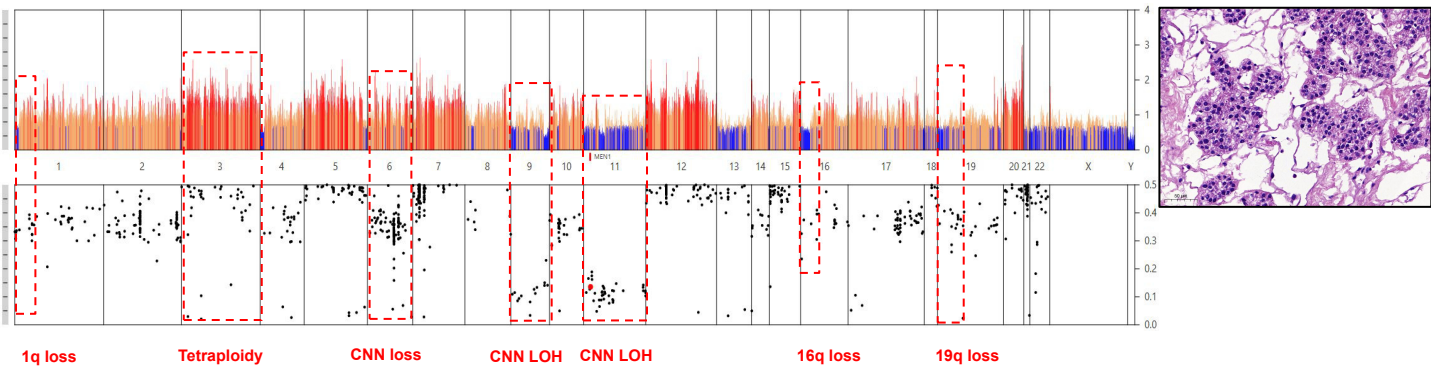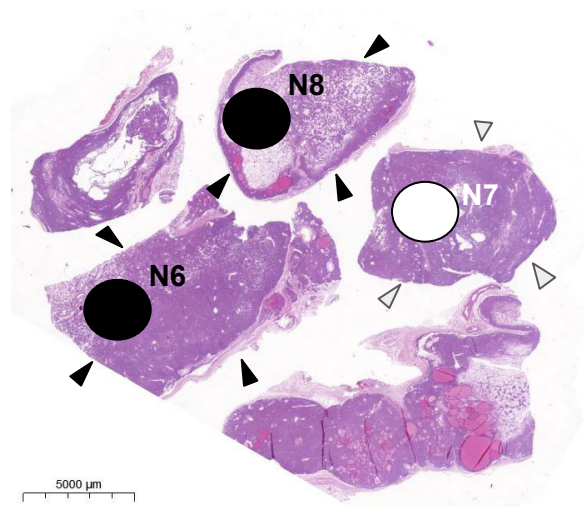

D  
N9

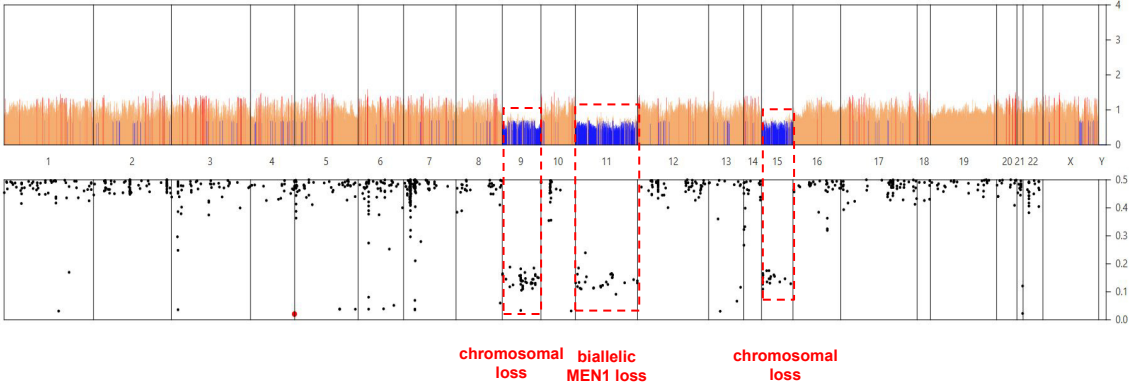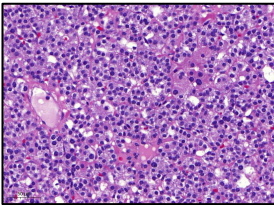

N10

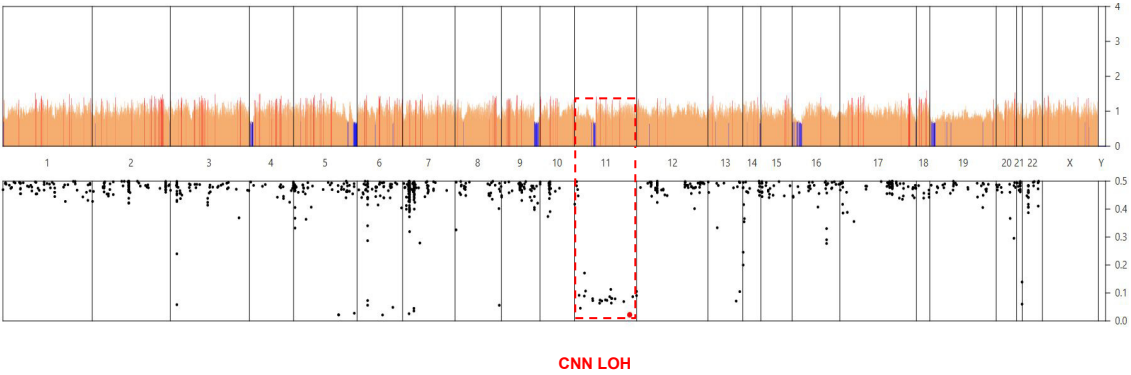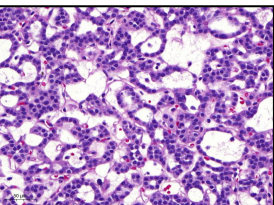

N11

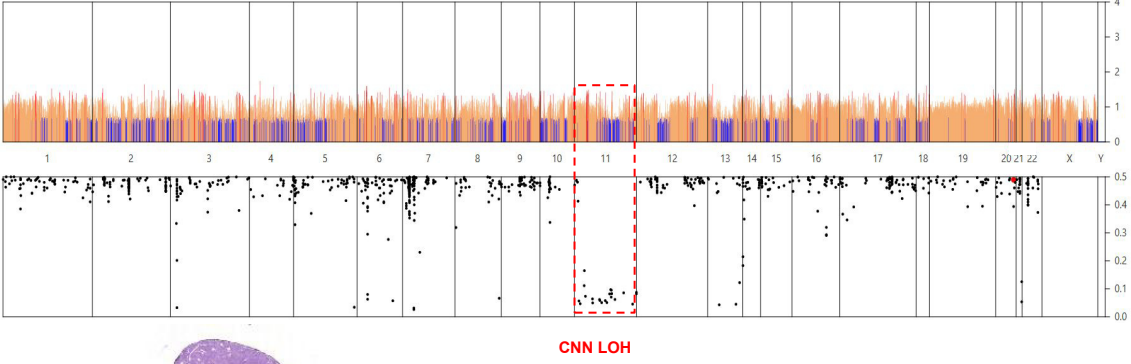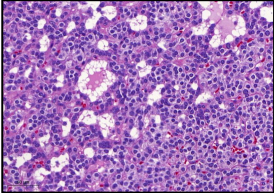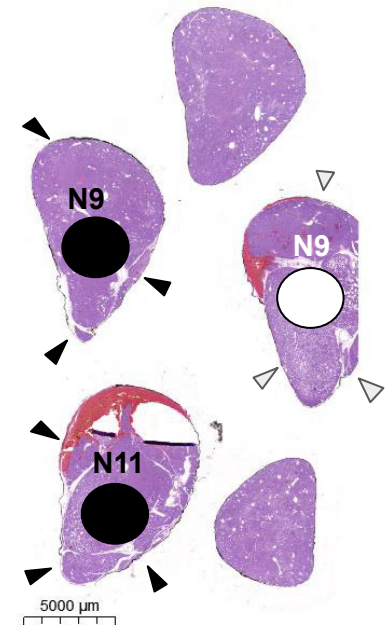

Supplement: Supplementary file 2 — Next-generation sequencing (NGS) of eleven nodules (“N”) in four MEN1 patients. A (patient “Vienna 16”, Table S1): N3 demonstrates biallelic MEN1 loss and is probably a different tumor, while N1 and N2 show conventional MEN1 LOH. N1 and N2 have different gains supporting subclonal evolution. B (patient “Vienna 9”): N4 and N5 demonstrate the same gains and losses, and represent most probably the same tumor. C (patient “Bern 1”): All three nodules share a CNN LOH of chromosome 11, as well as 16q and 19q losses. N6 and N8 have identical additional aberrations, suggesting N7 to be the primary clone with subclonal evolution in N6 and N8. Interestingly, N7 and N8 have cystic components. D (“Bern 4”): N9, N10 and N11 show different types of MEN1 inactivation supporting the concept of different tumors. In addition, N9 has further losses, N10 and N11 do not have. Bars (y-axis): number of reads; CNN: copy number neutral; LOH: Loss of heterozygosity; scatter plot: allelic frequency/single nucleotide polymorphisms (SNPs); x-axis: chromosomes (on the left q-arm, on the right p-arm). (PDF 7888 KB) [file 428_2023_3730_MOESM2_ESM.pdf]

**A**

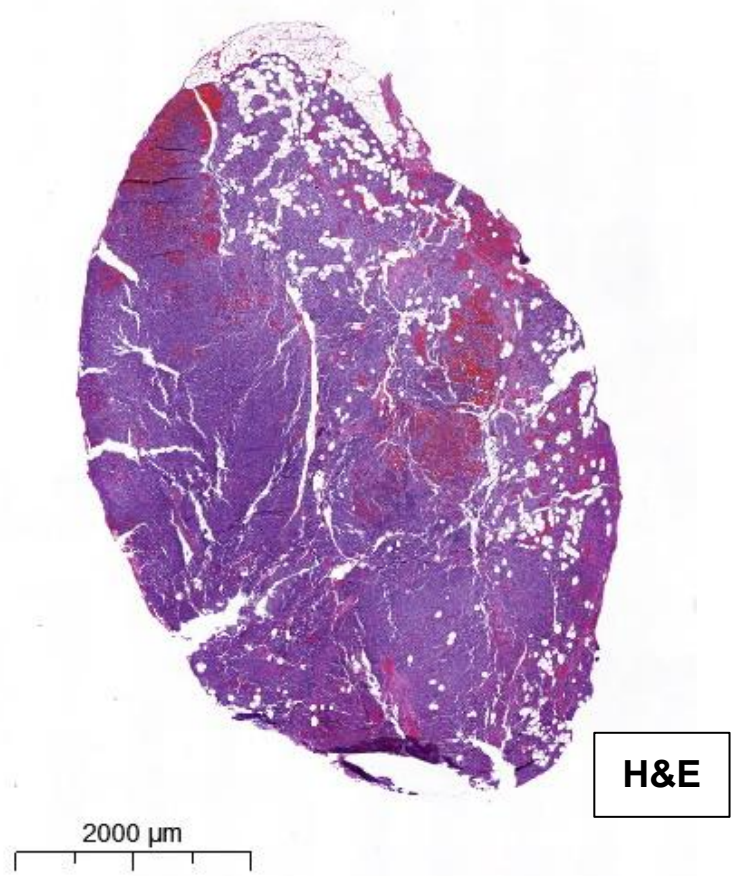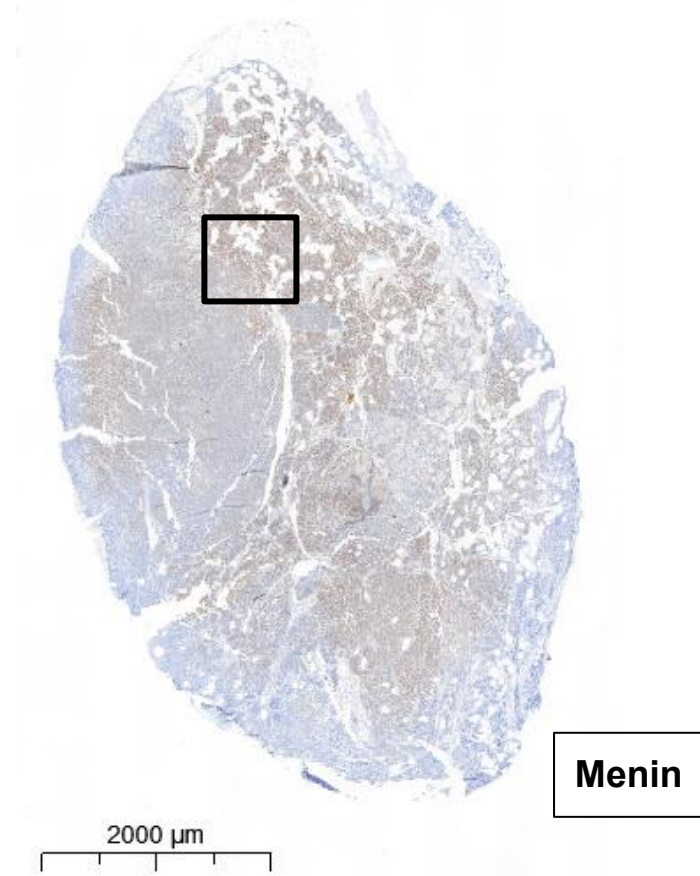

**B**

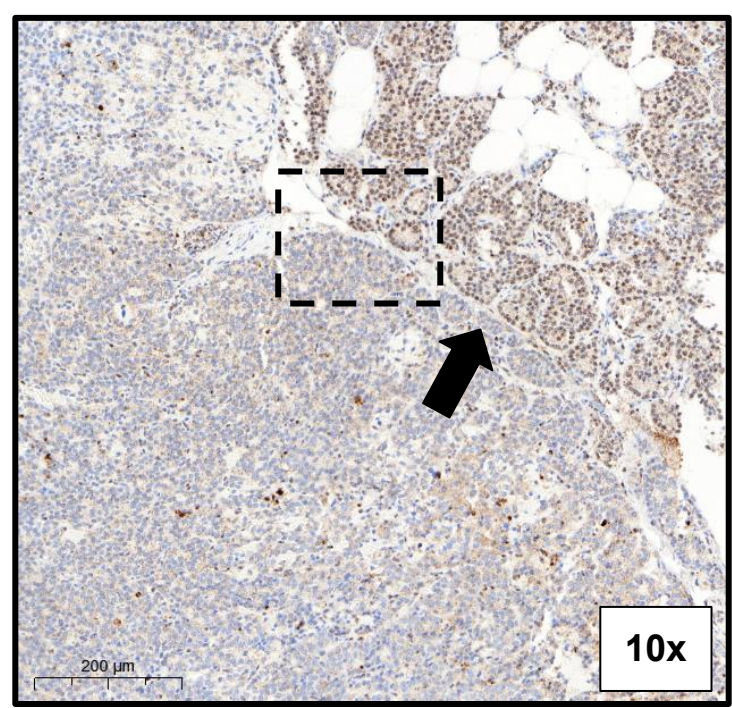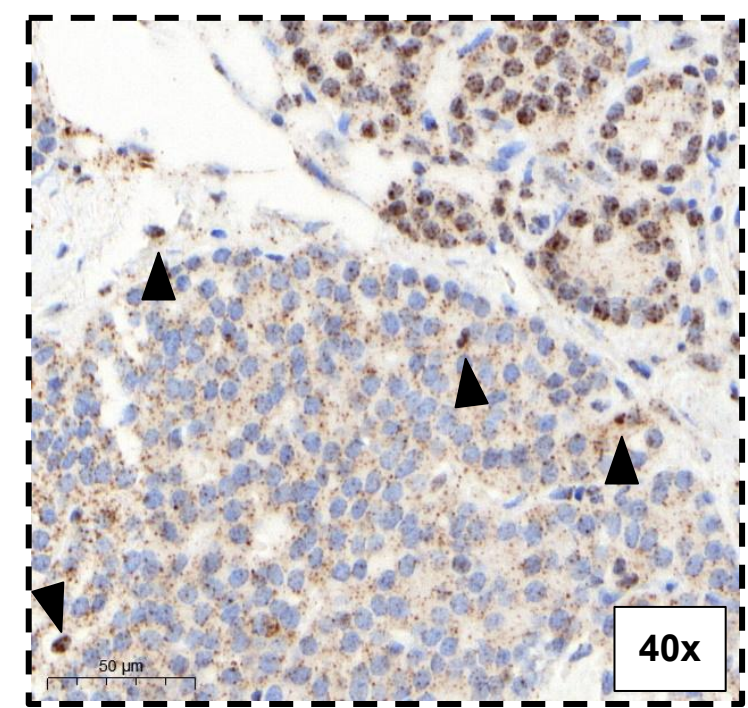

Supplement: Supplementary file 3 — Menin loss in a MEN1 parathyroid adenoma. A: MEN1 parathyroid adenoma (H&E), on the right Menin immunohistochemistry (overview). B: Loss of Menin expression in a larger clone, adjacent non-neoplastic parathyroid tissue (arrow) and intermingled non-neoplastic cells (arrowhead) with retained Menin expression. Inset: 40x magnification.(PDF 287 KB) [file 428_2023_3730_MOESM3_ESM.pdf]
