# Supplementary material for: Syndromic MEN1 parathyroid adenomas consist of both subclonal nodules and clonally independent tumors
Source: Virchows Arch. 2024 Jan 20;484(5):789–98. doi: 10.1007/s00428-023-03730-3 (PMC11106174; doi:10.1007/s00428-023-03730-3)
Supplement: Supplementary file 4 — (DOCX 16.1 KB) [file 428_2023_3730_MOESM4_ESM.docx]

**Table S1:** *MEN1* mutations. n/a: not available.

| **Patient** | **Year of surgery** | **Exon/intron** | **Mutation (original designation)** | **Menin expression** |
| --- | --- | --- | --- | --- |
| Vienna 1 | 1992 | Exon 3 | c.535G>A | non-evaluable |
| Vienna 2 | 1992 | Exon 3 | c.535G>A | non-evaluable |
| Vienna 3 | 1994 | Exon 4 | c.772C>T | non-evaluable |
| Vienna 4 | 1995 | Exon 9 | c.1213C>T | loss |
| Vienna 5 | 1996 | Exon 3 | n/a | non-evaluable |
| Vienna 6 | 2001 | Exon 2 | c.430T>G | non-evaluable |
| Vienna 7 | 2004 | Exon 9 | c.1243C>T | loss |
| Vienna 8 | 2005 | Exon 3 | c.496C>T | non-evaluable |
| Vienna 9 | 2007 | alternative Exon 2 | c.445+1 (cd149_150delTTGGfs151X) | loss |
| Vienna 10 | 2008 | alternative Exon 2 | c.445+1 (cd149_150delTTGGfs151X) | non-evaluable |
| Vienna 11 | 2009 | Exon 10 | c.1579C>T | loss |
| Vienna 12 | 2009 | Exon 10 | c.1398_1399delGfs91X | non-evaluable |
| Vienna 13 | 2010 | Exon 10 | c.1426_1430delGfs557X | non-evaluable |
| Vienna 14 | 2011 | Exon 8 | c.1059C>A | non-evaluable |
| Vienna 15 | 2016 | Intron 9 | c.1351-2A>G* | non-evaluable |
| Vienna 16 | 2016 | Exon 2 | c.1A>G | loss |
| Vienna 17 | 2017 | Exon 10 | c.1701_1705delCACCAinsTCG | non-evaluable |
| Vienna 18 | 2018 | Intron 4 | c.784-9G>A | loss |
| Vienna 19 | 2018 | Exon 1 | c.249_252del | loss |
| Vienna 20 | 2019 | Exon 10 | c.1378C>T | non-evaluable |
| Vienna 21 | 2019 | Intron 4 | c.784-9G>A | non-evaluable |
| Bern 1 | 2004 | Exon 9 | n/a | non-evaluable |
| Bern 2 | 2004 | Exon 10 | c.1378C>T | non-evaluable |
| Bern 3 | 2014 | Exon 3 | c.563G>C | loss |
| Bern 4 | 2022 | Exon 8 | c.1065-3C>G | loss |
